# Effectiveness of an app-based intervention to improve well-being through cultivating positive thinking and positive emotions in an adult sample: study protocol for a randomized controlled trial

**DOI:** 10.3389/fpsyg.2023.1200960

**Published:** 2023-07-19

**Authors:** Fabio Alexis Rincón Uribe, Maria Fernanda Monteiro Favacho, Paula Marília Nascimento Moura, Diana Milena Cortés Patiño, Janari da Silva Pedroso

**Affiliations:** ^1^Programa de Pós-Graduação em Psicologia, Universidade Federal do Pará, Belém, Pará, Brazil; ^2^Faculdade de Psicologia, Universidade Federal do Pará, Belém, Pará, Brazil; ^3^Programa de Pós-Graduação em Teoria e Pesquisa do Comportamento, Universidade Federal do Pará, Belém, Pará, Brazil

**Keywords:** well-being, positive emotions, positive thinking, positive psychology, mobile health, smartphone apps well-being, smartphone apps

## Abstract

**Introduction:**

Interventions to promote health and well-being based on the construction of psychological resources can positively impact the daily life of users and foster human flourishing. Nowadays, mobile health represents a safe way to support health research and implement evidence-based psychological interventions. The present study aims to evaluate the effectiveness of a mobile app-based intervention program (OneUS) designed to cultivate positive emotions and positive thinking to improve overall well-being.

**Methods:**

The study is designed as a randomized controlled trial. Two hundred participants will be randomly assigned to either a mobile health intervention condition (OneUS App) or an active control condition. The intervention consists of a program to cultivate positive emotions and positive thoughts. The active control intervention will comprise a set of mental imagery exercises and daily routine recording. The primary outcome comprises optimal well-being assessed using the multidimensional PERMA-Profiler scale. The sample will include adult people from the general population, who will be assessed at 8 time points: baseline (t1), intervention (t2, t3, t4, t5, t6) post-intervention (t7) and 3-month follow-up (t8).

**Discussion:**

Mobile apps seem to be promising tools to promote health and well-being. This study will evaluate the effectiveness of a mobile app (OneUS) aiming to cultivate positive emotions and positive thinking to improve well-being. The main strength of this study is the development of an evidence-based mobile health app, based on intentional mental training, to promote well-being. The limitations of this study relate to potential participant drop-out and the non-generalizability of the results to clinical populations.

**Clinical trial registration:**

https://ensaiosclinicos.gov.br/rg/RBR-43hpwqk, Identifier RBR-43hpwqk.

## Introduction

Well-being is a multidimensional construct that includes a hedonic and an eudaimonic component (Dahl et al., 2020). Hedonic well-being, often associated with feeling good, refers to experiencing more positive and less negative emotions, and higher levels of life satisfaction (Diener, 1984; Diener et al., 2017); whereas eudaimonic well-being, often associated with functioning well, includes self-acceptance, positive relationships, autonomy, mastery of the environment, purpose in life, and personal growth (Ryff, 1989; Martela and Sheldon, 2019). According to Seligman (2011), well-being is composed of five domains independently measured, represented in the acronym PERMA: Positive emotions (subjective measures of happiness and life satisfaction), Engagement (active involvement in activities), Relationship (positive connections with family, friends and colleagues), Meaning (the belief in something bigger than oneself) and, Accomplishment (motivation to fulfill personal goals and the satisfaction when those goals are achieved) (Butler and Kern, 2016). The combination of these domains contributes to fostering human flourishing and optimal life functioning at individual and collective levels (Seligman, 2011).

Research suggests that interventions to promote well-being that are focused on happiness, pleasure, and optimal psychological functioning are the most effective (Boiler et al., 2013; Weiss et al., 2016; Hendriks et al., 2020). A recent meta-analysis of 393 studies on different types of psychological interventions showed that interventions focused on building positive psychological resources (fortitude, gratitude, compassion, optimism) tend to have higher effect size estimates than those focused on reducing and controlling negative mental states (e.g., dysfunctional thoughts or psychopathological symptoms) (van Agteren et al., 2021). According to research, positive emotions and positive thinking help build personal or social resources that predict, over a lifetime, improvements in well-being ranging from increased hedonic experiences to increased psychological growth and life meaning (Kiken and Fredrickson, 2017; Coffey et al., 2022).

Positive emotions are multifaceted response tendencies that encompass physiological, subjective, cognitive, and behavioral aspects. They serve to broaden thoughts and actions in the present moment, leading to the development of long-lasting personal resources (Fredrickson, 2001). Positive emotions such as gratitude, hope, pride, love, and serenity can occur frequently in everyday life. Gratitude arises when individuals recognize someone or something as a source of good fortune. Hope emerges during challenging times when people aspire for the best outcomes. Pride arises when individuals attribute the fulfillment of their goals to their efforts. Love appears from meaningful interpersonal connections or relationships. Serenity is experienced when people perceive their circumstances as satisfactory, appreciated, or aligned with their values (Fredrickson, 2013). The experience of these positive emotions enriches well-being, and fosters greater social integration and satisfaction across various aspects of life, happiness, and resilience in the face of adversity (Fredrickson and Joiner, 2002; Silton et al., 2020).

On the other hand, positive thinking refers to a cognitive process that involves creating hopeful mental images, fostering optimistic beliefs, seeking advantageous problem-solving strategies, and cultivating an overall positive perspective on life (Bekhet and Zauszniewski, 2013). To develop positive thoughts, specific skills are employed, including transforming negative thoughts into positive ones, emphasizing the positive aspects of a situation, interrupting pessimistic thinking patterns, generating optimistic thoughts about problems, and devising methods to challenge negative thoughts. Positive thinking training emerges as a significant factor in enhancing well-being: empowering individuals to exert control over their life circumstances through positive appraisals of events and cultivating a pervasive optimistic perspective regarding their future, as well as the meaning and significance of life (Safari and Akbari, 2018; Chui and Chan, 2020).

Although the effectiveness of interventions to improve well-being has been demonstrated in controlled face-to-face settings, some difficulties and challenges may arise during the intervention process, such as non-attendance of participants, staff attrition, staff shortages, high costs, or laborious administration of assessment techniques and instruments (Griffiths et al., 2006). With the advent of new technologies and online tools, however, web-based intervention programs have emerged as a potential solution to these issues, offering advantages such as ease of access, rapid administration, and the ability for participants to engage in self-directed participation without the need for constant supervision (Marciniak et al., 2020; Szinay et al., 2020).

Mobile health (mHealth) is an example of the safe and cost-effective use of information and communication technologies (ICTs). The mHealth encompasses the use of mobile devices, such as cell phones, personal digital assistants, and other wireless devices, to support healthcare practices in various fields including medicine, psychology, and public health (World Health Organization, 2011). mHealth has contributed to increasing universal health coverage by providing timely information, decreasing overload on services, and enabling synchronization of care with daily lives of the users (Free et al., 2013; Marzano et al., 2015). To take advantage of these benefits, there are increasing efforts to design psychological interventions based on mobile applications to generate a positive impact on well-being and health (see Lorca-Cabrera et al., 2020 for a review): these interventions provide an accessible means to incorporate positive practices into existing routines and facilitate the adoption of new healthy habits (Diefenbach, 2018). Educational activities, along with techniques such as mindfulness, goal setting, future visualization, positive thought journaling, and mental imagery, might contribute to the success of technology-based well-being interventions (Howells et al., 2014; Riva et al., 2016; Eisenstadt et al., 2021).

The rapid advance of digital innovation has led to the availability of more than 350,000 health apps in major mobile app stores around the world. Among them, 47% are dedicated to well-being, according to data from IQVIA Institute for Human Data Science (2021). Despite the abundance of options, most apps lack adequate evaluations to determine their effectiveness in promoting and improving well-being (Donker et al., 2013). Although a recent meta-analysis suggests that health apps offer promising benefits in improving well-being, the overall effect size remains small based on pooled analysis (g = 0.17, 95% CI 0.05 to 0.29, *p* = 0.004) (Eisenstadt et al., 2021), and there is limited information on the long-term sustainability of these benefits beyond the intervention period (Boiler and Abello, 2014). Mobile apps are valuable tools that can provide quick and accessible benefits to many people; however, it is crucial that these apps are evidence-based to ensure their effectiveness and mitigate any potential adverse effects caused by their use.

Thus, a smartphone app named OneUS has been developed to support people in cultivating well-being through positive thinking and positive emotions (gratitude, hope, pride, love, and serenity). The app utilizes intentional mental training techniques and includes a library of psychoeducational content and guided exercises. By increasing awareness and teaching skills to cultivate positive emotions and train positive thinking in daily lives (Bekhet and Zauszniewski, 2013; Fredrickson, 2013), OneUs aims to enhance overall well-being. The app offers content in various formats, such as videos, audio, and text, which are enabled weekly after the completion of the visualization and activities. The app sends notifications and reminder messages to motivate users to continue the training, which will increase adherence rates.

This study aims to evaluate the effectiveness of a mobile app-based positive psychology intervention to improve people’s well-being through the daily practice of activities aimed at positive emotions and positive thinking. The study will test the hypothesis that using the OneUS mobile application for the daily practice of positive emotions and positive thinking enhances well-being and reduces psychological distress in a sample of adults from the general Brazilian population.

## Methods

### Study design

**Figure 1:**
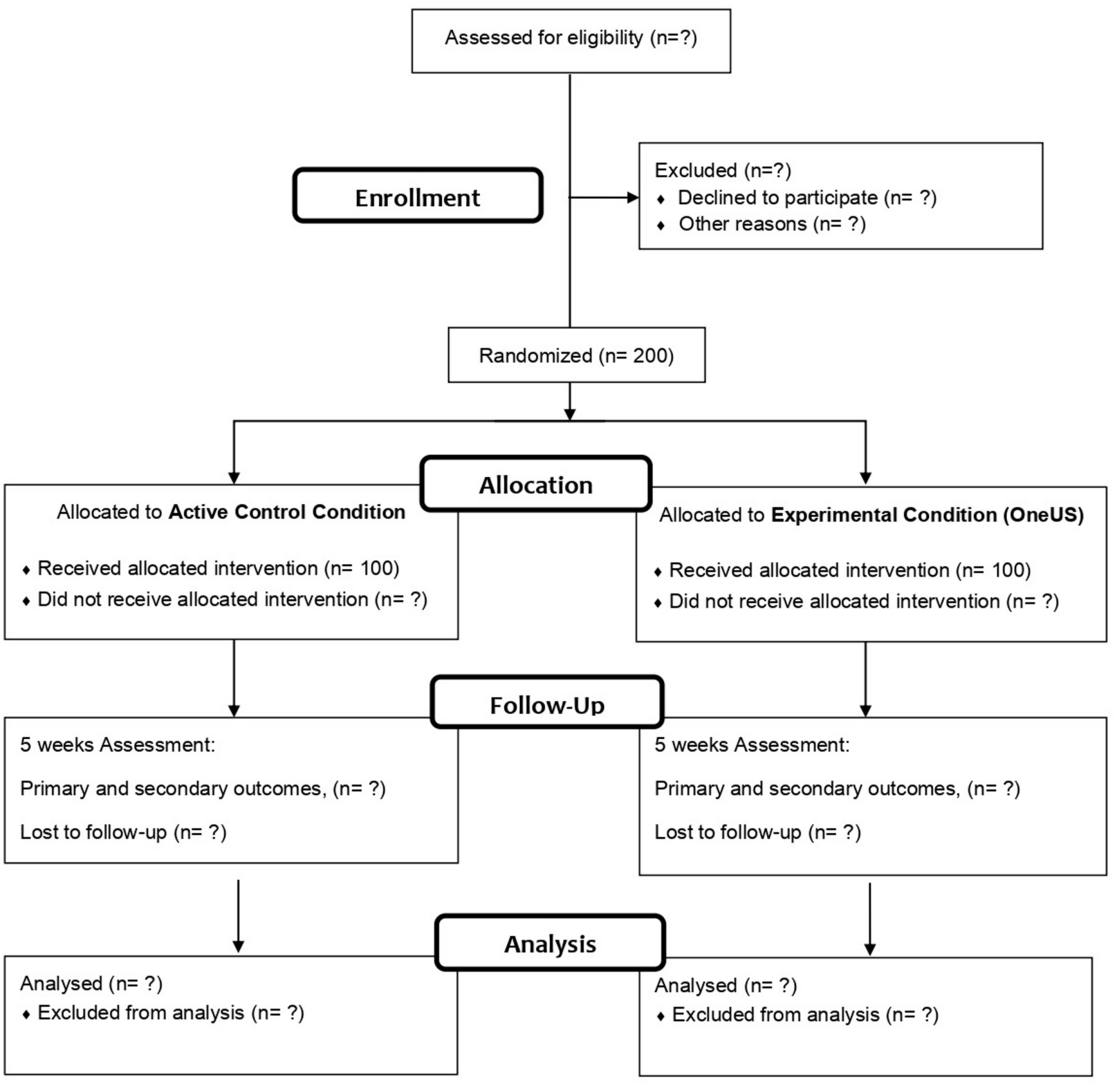
Stages of the study based on CONSORT diagram.

The protocol for this study received approval from the Research Ethics Committee of the Health Institute of the Federal University of Pará, Belém, Brazil (code number: CAAE: 58610822.1.0000.0018). The study was designed as a two-arm cluster-randomized controlled trial comparing an intervention condition and an active control condition. The stages of this study will include baseline assessment, implementation of the intervention, and weekly evaluation at seven measurement points. The stages of the study are shown in Figure 1. This study will be conducted in accordance with the Consolidated Standards of Reporting Trials (CONSORT) statement (Boutron et al., 2017) and the Consolidated Standards of Reporting Trials of Electronic and Mobile Health applications and online Telehealth (CONSORT-EHEALTH) checklist (Eysenbach and CONSORT-EHEALTH Group, 2011).

## Participants

### Sample size

This study will be conducted in Brazil. Participants will be recruited through advertising messages on social networks. In these messages, potential participants will find a link to a Google form to make an initial registration and provide their email addresses. The study aims to include 200 eligible participants, 100 per condition (active control and experimental). The General Linear Mixed Model Power and Sample Size 3.0 calculator (GLIMMPSE 3.0) (Kreidler et al., 2013) was used to determine the required sample size considering the study design, fixed predictors of conditions (active control and experimental), Time (t1, t2, t3, t4, t5, t6, t7, and t8), and Conditions x Time interaction effects. Using Hotelling Lawley Trace with power 0.8 and type I rate 0.05, total sample size was calculated as 76; however, 100 participants per condition will be selected to account for potential dropouts.

### Eligibility criteria

Participants will be selected through a step-by-step process that involves meeting certain eligibility criteria. To be eligible for inclusion in the study, participants must be adults (18 years or older), have access to the internet and a mobile device with an Android operating system, be a resident of Brazil, provide consent through an online form, agree to use the app, and complete the questionnaires. Individuals will be excluded if they have mental health problems. The General Health Questionnaire-12 (GHQ-12) will be utilized in this study to assess the risk of common mental disorders (CMD). It consists of 12 items and employs a Likert scale ranging from 1 to 4. A score of 1 indicates “More than usual,” while a score of 4 indicates “Much less than usual.” Each item on the questionnaire is scored as either absent (0) or present (1). Responses of 1 or 2 are considered absent (0), while responses of 3 or 4 are considered present (1). Participants who score positive for 3 or more items on the GHQ-12 will be classified as cases of CMD (Gouveia et al., 2003), and will be excluded from the study. In addition to the GHQ-12, two additional questions will be included to identify participants who have a diagnosis of psychological disorder or are currently undergoing psychological treatment for any mental health issues. The questions are as follows: “Do you have a diagnosis of a psychological disorder?” and “Are you currently receiving psychological treatment for any mental health issues?” Individuals who respond “Yes” to either of these questions will be excluded from the study.

### Recruitment

Potentially eligible participants who have pre-registered will be contacted by e-mail. In this message, they will be informed about the general aspects (i.e., rationale for the study, responsible institutions, benefits, procedures) and objectives of the study. In addition, they will be sent a link to access the App store to download the OneUS app for free. The recruitment of participants involves a self-selection process through the app. During this process, individuals will be required to respond to a series of questions that will be used to evaluate the selection criteria explained above. Based on their answers, the application will then determine whether to include or exclude them from the study. Noteworthy, the application incorporates a security mechanism designed to prevent access by bots and mitigate the risk of Distributed Denial of Service (DDoS) attacks. Upon installation, participants will be required to complete a pre-registration process, accept the terms, and verify their profiles using a code sent *via* email or text message. This procedure ensures that only human participants gain access to the application, safeguarding and guaranteeing the security of their personal data and the progress achieved in the mobile application.

## Procedures

### Randomization and blinding

Participants will be randomly assigned to either the intervention condition or the active control condition using R software (Version 4.0.5) and the R package randomizr. The allocation ratio will be 1:1. The randomizr function, applied by a blinded research assistant, will randomly assign a value of 0 or 1 to each participant, representing their experimental condition (0 = Experimental; 1 = Active Control). Although participants and evaluators will not be blinded to the intervention during the study, the data analysts will be blinded. Participants may be able to determine which condition they are in due to the educational and informative content they receive about positive emotions and positive thinking.

### Interventions

OneUs is an application developed by the authors in collaboration with Educa Tecnologia, a Brazilian company that provided computer programming services. The App has two sub-applications: an intervention app and a control app. Once potential participants are selected, they will be randomly assigned to one of these sub-applications.

### Experimental condition: OneUS intervention app

The intervention app is a 5-week program comprising 11 training sessions specifically designed to cultivate and enhance and positive emotions (gratitude, hope, pride, love, and serenity) and positive thinking. The program includes one introductory session with educational content on positive emotions and positive thinking, five sessions to cultivate positive emotions and five sessions for training the skills necessary to create and maintain positive thoughts. Each week, the program will focus on simultaneously practicing a specific positive emotion and a positive thinking skill (refer to Table 1). These practices will include educational activities (such as psychoeducational videos and informational content), assessment questions (to evaluate knowledge about positive thinking and positive emotions), feedback exercises (including identifying, modifying, and cultivating positive thoughts and expressing positive emotions), and guided or self-administered practice (such as mindfulness and Love-Kindness Meditation). The app (OneUS) will monitor individual progress by tracking content consumption and exercise performance, and participants will receive notifications and messages as reminders to engage in each session.

**Table 1:** General content and weekly activities.

		Intervention weeks				
		Week 1	Week 2	Week 3	Week 4	Week 5
Positive emotions	Content	Gratitude	Authentic pride	Love	Hope	Serenity
	Daily practice	Counting blessings	Reflective meditation and self-affirmation messages	Loving-kindness Meditation	Brief Hope Intervention	Mindfulness exercise
Positive thinking	Content	Recognizing negative and positive thoughts	Turn negative thoughts into positive ones	Identify the positive aspects of everyday situations	Problem-management	Interrupting negative thoughts
	Daily practice	Journal of thoughts	Positive self-talk	Recording of experiences and positive thinking	Positive internal dialog	Best possible self

In the introductory session, information on the advantages of cultivating positive emotions and positive thinking will be presented (MacLeod and Moore, 2000; Kok and Fredrickson, 2013). In the first week, the practice of positive emotions will involve a daily exercise of counting blessings, where participants identify and record things, they are grateful for Boggiss et al. (2020). The positive thinking activity will involve acknowledging both positive and negative thoughts and documenting them in a journal.

In the second week, the practice of *positive emotions* will focus on cultivating authentic pride through reflective meditation and self-affirmation messages, which will increase awareness of accomplishments, motivations, and successful outcomes resulting from the participant’s actions (Hill et al., 2020). The *positive thinking* activity will involve learning how to turn negative thoughts into positive ones. The practice will involve strengthening of positive self-talk to organize thoughts, assign positive meanings, and engage in silent or verbal self-dialog (Santos-Rosa et al., 2022).

The third week will focus on cultivating *positive emotions* by introducing the concept of love - which can create a feeling of connection and motivation with oneself and others. Loving-kindness meditation (LKM) will be used to strengthen unconditional expressions of love and care for self and others, and to foster an altruistic desire for success, happiness, and a positive future for all (Zeng et al., 2015). The guided practice of LKM is based on a tool designed by Seppala et al. (2014). The *positive thinking* practice will focus on identifying the positive elements of situations or events of everyday life (e.g., accomplishing a goal, successfully completing an everyday task, recognizing a personal skill), regardless of whether they are favorable or adverse. Participants will keep a daily log of their experiences and recognize any positive thoughts around these events that arise naturally (Rice and Fredrickson, 2016).

During the fourth week, *positive emotion* practice will focus on cultivating hope through a condensed version of the Brief Hope Intervention (BHI) (Chan et al., 2010). The BHI will be adapted to a virtual environment to promote hopeful internal dialogs about goals (goal setting), pathways to reach goals, and strategies to overcome obstacles (Snyder et al., 2000). The *positive thinking* activity will consist of learning to manage problems by breaking them into smaller parts and creating a positive internal dialog about each part (Naseem and Khalid, 2010).

In the fifth week of the program, the practice of *positive emotions* will encompass the cultivation of serenity, with a guided mindfulness exercise to become aware of current favorable or difficult situations and elicit feelings of peace and acceptance of the experiences evoked (Zoellner et al., 2007). The *positive thinking* practice will consist of interrupting negative thoughts through relaxation exercises. These exercises will include brief guidance on abdominal breathing, progressive muscle relaxation, and Mindfulness (Toussaint et al., 2021). In addition, there will be a guided practice of the best possible self, which will contain visualization exercises of oneself reaching one’s full potential and generating positive thoughts from this (Carrillo et al., 2019).

### Active control condition

The comparator will be an active control condition. Participants in this condition will be trained in mental imagery exercises in normal everyday life situations. This condition will be considered an active control because the participants will perform mental imagery of neutral events and emotion monitoring. The app design for this condition will have the same structure as the experimental condition and will include a user profile, menu, assessment measures, and notifications.

## Outcome measures

The outcome measures will be completed at 8 time points: baseline (t1), intervention (t2, t3, t4, t5, t6) post-intervention (t7), and 3-month follow-up (t8), following the recommended timeline outlined in the SPIRIT 2013 statement (refer to Table 2). The SPIRIT 2013 statement serves as a checklist for clinical trial protocols, offering guidelines on the essential content and a standardized diagram of activities, interventions, and measures (Chan et al., 2013).

**Table 2:** Schematic diagram for timeline participant.

	Enrolment	Baseline	Intervention					Close-out	Follow-up
TIMEPOINT	*0*	*t1*	Week 1 (t2)	Week 2 (t3)	Week 3 (t4)	Week 4 (t5)	Week 5 (t6)	*t_7_*	*t8*
Enrolment:									
Eligibility screen	X								
Informed consent	X								
Allocation	X								
Interventions:									
Training and daily practice of positive emotions and positive thinking			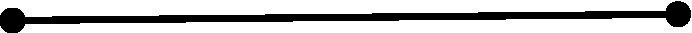						
Active control			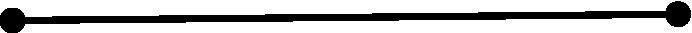						
Assessments:									
Socio-demographic characteristics	X								
Well-being		X	X	X	X	X	X	X	X
Optimism		X	X	X	X	X	X	X	X
Hope		X	X	X	X	X	X	X	X
Subjective Happiness		X	X	X	X	X	X	X	X
Stress		X	X	X	X	X	X	X	X

## Primary outcome

### Well-being

Well-being will be assessed with the PERMA-profiler Scale (Butler and Kern, 2016). This scale evaluates well-being across five dimensions: Positive Emotions, Engagement, Relationships, Meaning, and Accomplishment. Additionally, it includes measures for physical health, negative emotions, loneliness, and overall happiness. The scale consists of 23 items on an 11-point scale, that are scored by calculating the arithmetic mean. The closer the mean is to 11, the higher the levels of well-being. The PERMA-profile was adapted and validated for use in the Brazilian population using a sample of 1,327 adults. The results indicated reliable indices for the overall PERMA factor (Guttman λ6 = 0.94 and McDonald Ω = 0.94), and for each individual dimension: Positive Emotion (λ6 = 0.86 and Ω = 0.90), Engagement (λ6 = 0.52 and Ω =. 62), Relationships (λ6 = 0.71 and Ω = 0.78), Meaning (λ6 = 0.80 and Ω = 0.86), and Accomplishment (λ6 = 0.76 and Ω = 0.81) (De Carvalho et al., 2021).

### Secondary outcomes

#### Perceived stress

The perception of stress will be evaluated with the Perceived Stress Scale (PSS-10). This scale evaluates whether a person perceives everyday situations as stressful (Cohen et al., 1983). It consists of 10 items with response options that range from “Never” to “Very often,” higher scores indicate greater perceived stress. The PSS-10 was adapted and validated for use in the Brazilian population using a sample of 793 adults and showed adequate indices of reliability, with an alpha of 0.83 (Reis et al., 2010).

#### Optimism

Optimism will be assessed with the Life Orientation Test (LOT-R). The LOT-R is a self-report test that uses a Likert scale to measure optimism as a single trait. The test comprises 10 items, with six items formulated positively (items 1, 4, 10) and negatively (items 3, 7, 9), and four distractor items (2, 5, 6, 8). Participants are required to indicate their level of agreement with each item on a 5-point scale, where 1 represents “strongly disagree” and 5 represents “strongly agree.” The total score can range from 6 to 30. To calculate the score, the values of the negative elements are reverse scored and then added to the values of the positive elements, with higher scores indicating greater optimism. The LOT-R was adapted and validated for use in the Brazilian population using a sample of 844 adults and showed adequate internal reliability indices, with an alpha of 0.80 (Bastianello et al., 2014).

#### Hope

Hope will be measured by the Dispositional Adult Hope Scale (DHS), a self-report test consisting of 12 items that measure two dimensions of hope: Agency (4 items) and Means (4 items), with four items as distractors (Snyder et al., 1991). Respondents will rate their agreement with each item on an eight-point Likert scale ranging from 1 (definitely false) to 5 (definitely true). Scores are calculated by adding up the ratings across all items, with higher scores indicating greater levels of hope. A Brazilian adaptation of the DHS was validated using a sample of 844 Brazilian adults and showed adequate indices of reliability, with an alpha of 0.79 (Hutz, 2014).

#### Happiness

Happiness is determined using the Subjective Happiness Scale (SHS), which provides a subjective assessment of an individual’s overall happiness or unhappiness (Lyubomirsky and Lepper, 1999). The SHS consist of four items and seven response options ranging from 1 to 7. Scores between 4 and 28 are possible, with higher scores indicating higher levels of happiness. The SHS was adapted and validated for use in the Brazilian population in a study with 600 Brazilian adults, and it showed a satisfactory reliability index, with an alpha of 0.82 (Damásio et al., 2014).

## Data collection and management

Data will be collected online via the OneUS app. A sociodemographic questionnaire and measures of well-being, optimism, hope, happiness, and stress will be used. Data will be stored in an online database. Data collected online will be automatically stored on a local server (CentOS Linux Dedicated on Google Cloud Platform). In accordance with the LGPD (Brazilian General Data Protection Law), it is necessary to ensure the rights to freedom and privacy when processing personal data of participants, regardless of whether it is in digital or physical environments. To ensure accuracy and minimize errors, a researcher will periodically review and verify the input and accuracy of the data that is being entered. To improve adherence to the intervention, participants will receive notifications and motivational messages *via* app and email. In addition, the app has a feature that will inform users of their participation level and remind them to continue their training when the weekly participation rate is below the desired level.

## Data analysis methods

Statistical methods for primary and secondary outcomes.

The analysis will be conducted using linear mixed models. This statistical approach is well-suited for handling the structure of the repeated measures inherent in our experimental design, enabling us to model the fixed effects of conditions (OneUS app intervention and Control active) and time (t1, t2, t3, t4, t5, t6, t7, and t8) and Conditions x Time interaction effects (DeHart and Kanpla, 2019). We will conduct data analysis using Maximum Likelihood Estimate (MLE) to calculate model parameters. This method allows for simultaneous estimation of all model parameters, including the variance of random effects. To ensure the validity of the model, we will verify the normality of the residuals. Hypothesis tests will be conducted to assess the significance of the fixed effects. The analyses will be carried out using the R statistical language (R Core Team, 2023) in the RStudio environment, utilizing the most up-to-date versions of the lme4 packages at the time of analysis.

## Discussion

The present study aims to evaluate the effectiveness of a mobile app (OneUS) based on positive psychology intervention to improve people’s well-being, through the daily practice of positive emotions (gratitude, hope, pride, love, and serenity) and positive thinking. The intervention is a five-week program that will include awareness exercises, training, and guided practice of positive emotions and positive thinking.

Although mobile app-based interventions have great potential to improve well-being, evidence supporting their efficacy remains limited (Eisenstadt et al., 2021). With the proliferation of numerous well-being apps, studies evaluating the effectiveness of these interventions are needed. Particularly valuable are studies that assess post-intervention benefits, as they provide insight into the durability of the effects achieved and identify the characteristics of users who benefit most from such interventions. For instance, a comprehensive review of two large longitudinal data sets revealed that individuals who participated in online interventions were able to maintain their levels of well-being for several months. In particular, those who initially reported lower levels of well-being and continued to actively participate were more likely to experience lasting positive outcomes (Sanders et al., 2019).

The main strength of the present study lies in the development of an evidence-based mobile health application aimed at promoting well-being. The use of a randomized controlled trial design minimizes bias and provides a rigorous means of assessing the impact of the intervention. The intervention is based on intentional mental training, a psychological mechanism that has been shown to be highly effective in improving well-being (Dahl et al., 2020). Through coaching, feedback exercises and daily practices, this mobile-based intervention could have a direct implication on daily lives of the participants by increasing their positive emotions and positive thinking, potentially improving their overall well-being. On the other hand, the present study has the following limitations: healthy participants from the general population will be included, so the results obtained cannot be generalized to clinical populations. Another limitation is the lack of an effective adherence strategy to ensure the completion of the training by the participants. However, in order to increase the adherence rate to the program, notifications and reminder messages will be sent by the app and *via* email to motivate the participant to continue with the training. Also, the number of participants was expanded to overcome the dropout rate during the intervention.

## Ethics statement

The study was approved by the Research Ethics Committee of the Instituto de Ciências da Saúde da Universidade Federal do Pará, Belém, Brazil with registration number CAAE:58610822.1.0000.0018. To participate in the study, informed consent will be obtained from participants through the app.

## Author contributions

FU is the principal investigator, conceived the study, led the proposal, protocol development, and drafting of the manuscript. PM contributed to the study design and proposal development. MF contributed to the creation of the new software (App) to be used in the study. DP contributed to the methodological design and to the review and editing of the manuscript. JS was the lead methodologist for the trial. All authors contributed to the article and approved the submitted version.

## Funding

This trial is funded by Universidade Federal do Pará, Brasil/ Pró-Reitoria de Pesquisa e Pós-graduação (PROPESP), Coordenação de Aperfeiçoamento de Pessoal de Nível Superior – CAPES (Grant number 88887.657744/2021-00), and Mineração Paragominas S/A - Norsk Hydro Brasil (Grant number 4444).

## Conflict of interest

The authors declare that the research was conducted in the absence of any commercial or financial relationships that could be construed as a potential conflict of interest.

## Publisher’s note

All claims expressed in this article are solely those of the authors and do not necessarily represent those of their affiliated organizations, or those of the publisher, the editors and the reviewers. Any product that may be evaluated in this article, or claim that may be made by its manufacturer, is not guaranteed or endorsed by the publisher.
